# SCOTROC 2B: feasibility of carboplatin followed by docetaxel or docetaxel–irinotecan as first-line therapy for ovarian cancer

**DOI:** 10.1038/sj.bjc.6602910

**Published:** 2005-12-13

**Authors:** A R Clamp, J Mäenpää, D Cruickshank, J Ledermann, P M Wilkinson, R Welch, S Chan, P Vasey, B Sorbe, A Hindley, G C Jayson

**Affiliations:** 1 Cancer Research UK Department of Medical Oncology, Christie Hospital, Manchester M20 4BX, UK; 2 Department of Obstetrics and Gynaecology, Tampere University Hospital, FIN-33521 Tampere, Finland; 3 Women and Children's Directorate, James Cook University Hospital, Middlesbrough TS4 3BW, UK; 4 Department of Oncology, University College London, London W1P 8BT, UK; 5 Department of Clinical Oncology, Christie Hospital, Manchester M20 4BX, UK; 6 Nottingham City Hospital, Nottingham NG5 1PB, UK; 7 Division of Oncology, Cancer Care Services, Royal Brisbane and Women's Hospital, Herston, Queensland 4029, Australia; 8 Department of Gynecological Oncology, Örebro University Hospital, SE-701 85 Örebro, Sweden; 9 Rosemere Cancer Centre, Royal Preston Hospital, Fullwood, Preston PR2 9HT, UK

**Keywords:** taxane, docetaxel, carboplatin, irinotecan, ovarian, cancer

## Abstract

The feasibility of combination irinotecan, carboplatin and docetaxel chemotherapy as first-line treatment for advanced epithelial ovarian carcinoma was assessed. One hundred patients were randomised to receive four 3-weekly cycles of carboplatin (area under the curve (AUC) 7) followed by four 3-weekly cycles of docetaxel 100 mg m^−2^ (arm A, *n*=51) or docetaxel 60 mg m^−2^ with irinotecan 200 mg m^−2^ (arm B, *n*=49). Neither arm met the formal feasibility criterion of an eight-cycle treatment completion rate that was statistically greater than 60% (arm A 71% (90% confidence interval (CI) 58–81%; *P*=0.079; arm B 67% (90% CI 55–78%; *P*=0.184)). Median-dose intensities were >85% of planned dose for all agents. In arms A and B, 15.6 and 12.2% of patients, respectively, withdrew owing to treatment-related toxicity. Grade 3–4 sensory neurotoxicity was more common in arm A (1.9 *vs* 0%) and grade 3–4 diarrhoea was more common in arm B (0.6 *vs* 3.5%). Of patients with radiologically evaluable disease at baseline, 50 and 48% responded to therapy in arms A and B, respectively; at median 17.1 months’ follow-up, median progression-free survival was 17.1 and 15.9 months, respectively. Although both arms just failed to meet the formal statistical feasibility criteria, the observed completion rates of around 70% were reasonable. The addition of irinotecan to first-line carboplatin and docetaxel chemotherapy was generally well tolerated although associated with increased gastrointestinal toxicity. Further exploratory studies of topoisomerase-I inhibitors in this setting may be warranted.

Ovarian cancer is the fourth most commonly occurring cancer in women, with most patients presenting with extra-pelvic disease spread. Treatment of this advanced disease (International Federation of Gynecologic Oncology (FIGO) stage III–IV) involves cytoreductive surgery followed by chemotherapy. Based on the results of the pivotal GOG-111 and OV-10 studies, which demonstrated a survival advantage for cisplatin–paclitaxel over cisplatin–cyclophosphamide (McGuire *et al*, 1996; Piccart *et al*, 2000), first-line chemotherapy usually includes a platinum agent plus paclitaxel. However, median survival in these studies was only 3 years and an estimated 130 000 deaths per year still occur from ovarian cancer worldwide (Shibuya *et al*, 2002). Paclitaxel treatment is also associated with peripheral neuropathy (Guastalla and Dieras, 2003). Notably, the second-generation taxane docetaxel in combination with carboplatin is as effective as carboplatin–paclitaxel for first-line therapy (Vasey *et al*, 2004), with myelosuppression rather than peripheral neuropathy being the predominant toxicity.

One method for improving outcomes may be to incorporate a third cytotoxic agent into first-line therapy – specifically, a new potentially non-cross-resistant agent with demonstrated activity in relapsed disease. Promising agents include the topoisomerase-I inhibitors topotecan and irinotecan, which are active in platinum-resistant ovarian cancer (Takeuchi *et al*, 1991; Gordon *et al*, 2001; Bodurka *et al*, 2003). Irinotecan has also exhibited activity in combination with docetaxel at a recommended 3-weekly dose of irinotecan 200 mg m^−2^ and docetaxel 60 mg m^−2^ (Mäenpää *et al*, 1998). A response rate of 63% was reported with docetaxel–irinotecan treatment for recurrent disease (Mäenpää *et al*, 2002); the predominant toxicities were neutropenia and diarrhoea (Mäenpää *et al*, 1998, 2002).

The use of triplet cytotoxic combinations, however, can be complicated by significant toxicity – in particular, myelosuppression, which impacts on dose intensity. One means of circumventing this is to administer agents sequentially, as has been performed successfully in the adjuvant treatment of breast cancer (Bonadonna *et al*, 1995). The results of the GOG-132 study suggest that such an approach could also be used in ovarian cancer (Muggia *et al*, 2000). In that trial, chemonaïve patients with advanced ovarian cancer received either combination cisplatin–paclitaxel or each of these drugs alone. However, as many patients receiving single-agent therapy were subsequently crossed-over to the other drug before clinical evidence of disease progression, they were essentially receiving sequential therapy. No survival differences were detected between the three arms, suggesting that sequential therapy is as effective as the concurrent approach.

Laboratory data also indicate that sequential therapy may be appropriate using platinum and taxane drugs. For instance, cell lines with p53 mutations are hypersensitive to paclitaxel but resistant to platinum, whereas cells with normal p53 function are platinum sensitive (Wahl *et al*, 1996). Hence, initial platinum treatment might eradicate a population of wild-type p53 tumour cells, leaving a population of predominantly mutant p53 cells that are amenable to subsequent treatment with taxanes. Thus, sequential administration of optimal doses of individual agents may allow maximum impact on distinct chemosensitive cell populations, while avoiding the toxicity problems commonly associated with concurrent combination chemotherapy.

This study investigated the feasibility of sequential carboplatin followed by docetaxel or docetaxel–irinotecan as first-line therapy in patients with FIGO stage Ic–IV epithelial ovarian, fallopian tube or primary peritoneal carcinomas.

## PATIENTS AND METHODS

### Patient population

Eligible patients were women aged ⩾18 years with a histopathological diagnosis of invasive epithelial ovarian carcinoma (EOC), fallopian tube cancer or ovarian-type primary peritoneal cancer. Other inclusion criteria were as follows: FIGO disease stage Ic–IV (stage Ic patients were limited to those with malignant cells in ascitic fluid, peritoneal washings or with tumour on the surface of the ovary); Eastern Cooperative Oncology Group performance status of 0–2; no prior chemotherapy or radiotherapy; adequate bone marrow (neutrophils >1.5 × 10^9^ l^−1^ or platelets >100 × 10^9^ l^−1^, haemoglobin >9.0 g dl^−1^), renal (serum creatinine <1.25 × upper limit of normal (ULN)) and hepatic function (bilirubin <1 × ULN, aspartate aminotransferase/alanine aminotransferase <1.5 × ULN and alkaline phosphatase <2.5 × ULN).

Exclusion criteria included the following: borderline or mixed mesodermal tumours; previous or concurrent malignancy within the preceding 5 years (except curatively treated uterine cervical carcinoma *in situ* or basal cell skin carcinoma); concurrent severe and/or uncontrolled comorbidities; prior serious allergic reactions; ongoing bowel obstruction or current inflammatory bowel disease or chronic diarrhoea; pregnancy or lactation; symptomatic peripheral neuropathy ⩾grade 2. A maximum of 8 weeks was permitted between initial surgery and starting trial chemotherapy.

All patients gave written informed consent. The trial was approved by the Manchester Ethics Committee and by the local ethics committees of the other participating hospitals.

### Treatment

All patients initially received four cycles of carboplatin AUC 7 (AUC=area under the curve, calculated using the Calvert formula (Calvert *et al*, 1989) where the glomerular filtration rate (GFR) was measured using an isotopic method). Carboplatin was infused over 1 h in 500 ml of 5% dextrose every 3 weeks. After carboplatin initiation treatment, arm A patients received docetaxel 100 mg m^−2^ (1-h infusion) every 3 weeks for four cycles and arm B patients received docetaxel 60 mg m^−2^ (1-h infusion) followed ⩾30 min later by irinotecan 200 mg m^−2^ (30-min infusion) every 3 weeks for four cycles.

Docetaxel premedication comprised 8 mg oral dexamethasone twice daily for 3 days, commencing 24 h before docetaxel administration in both arms. No specific premedication for irinotecan was given. If symptoms suggestive of an acute cholinergic syndrome developed during treatment, atropine (0.25 mg subcutaneous) was recommended and given prophylactically with subsequent cycles. High-dose loperamide was administered for delayed diarrhoea and fluoroquinolone antibiotics if diarrhoea occurred in the context of fever or grade 3–4 neutropenia.

### Dose and schedule modification

On each scheduled treatment day, full doses of all drug(s) were administered only if neutrophils were ⩾1.5 × 10^9^ l^−1^ and platelets ⩾100 × 10^9^ l^−1^; otherwise, a treatment delay of ≤2 weeks was permissible. Haematological recovery after 1 week resulted in the full dose being given at the next treatment cycle. If treatment was delayed by >1 week, or was complicated by neutropenic fever or grade 4 thrombocytopenia, the dose was reduced to dose level –1 (Table 1). Recurrence of significant haematological toxicity with subsequent cycles resulted in a further reduction to dose level –2. A delay of >2 weeks for haematological recovery or further significant haematological toxicity after two dose reductions necessitated patient withdrawal from the study.

Prophylactic oral antibiotics were administered with subsequent cycles in patients with grade 4 neutropenia accompanied by a single oral temperature of >38.5°C. Routine use of haematopoietic growth factors was not recommended. If serum creatinine increased to >1.25 × ULN with carboplatin, the GFR was remeasured. A fall in GFR of >25% from baseline resulted in patient withdrawal from protocol therapy. The development of grade 3–4 mucositis or hepatic toxicity during docetaxel chemotherapy necessitated treatment delay until resolution of toxicity to <grade 2 and subsequent cycles were administered at dose level –1.

For patients receiving irinotecan, the occurrence of grade 3–4 diarrhoea (despite appropriate management) led to a 25% dose reduction of irinotecan with subsequent cycles. If significant diarrhoea recurred, the docetaxel dose was also reduced by 25% and treatment was discontinued if this toxicity still persisted. All patients experiencing grade 3–4 neurotoxicity or significant cardiac toxicity were withdrawn from treatment.

### Clinical assessments

A full clinical examination (including pelvic examination) and electrocardiography were performed at baseline. Clinical examination was repeated before each treatment cycle. Laboratory investigations at baseline incorporated a full blood count (FBC; including differential white cell count), full biochemical profile and CA-125. The FBC and full biochemical profile were repeated on the day of chemotherapy administration during carboplatin chemotherapy, and weekly during docetaxel-based chemotherapy. CA-125 measurement was repeated with each treatment cycle.

Disease extent at baseline was established by abdominopelvic computed tomography (CT) scan and chest radiograph. Radiological assessment was repeated after cycles 4 and 8 if evaluable disease was present on baseline imaging or if there was clinical or CA-125 evidence of disease progression. If interval debulking was performed after carboplatin chemotherapy, repeat imaging was required before starting docetaxel-containing chemotherapy. Disease response was assessed using modified Southwest Oncology Group criteria. Patients with disease progression after completing the carboplatin phase proceeded to the docetaxel-based treatment phase (unless the treating clinician deemed this inappropriate).

Patient quality of life (QoL) was assessed at baseline using the European Organisation for Research and Treatment of Cancer (EORTC) core questionnaire QLQ-C30 combined with ovarian cancer module QLQ-OV28. These questionnaires were repeated after completion of carboplatin treatment and before each docetaxel-based chemotherapy cycle. Toxicities were documented throughout chemotherapy using the National Cancer Institute of Canada Expanded Common Toxicity Criteria (version 2.0).

Patient follow-up – including full clinical and pelvic examinations, serum CA-125 measurement and QoL assessment – was performed every 2 months until disease progression or for a maximum of 2 years. Abdominopelvic CT scan was performed if progressive disease was suspected.

### Statistical considerations

This was a multicentre, prospective, two-arm, randomised phase II feasibility study. Treatment allocation was performed by minimisation with stratification for centre, FIGO stage, performance status and residual disease. The primary end point was the percentage of patients completing eight cycles of chemotherapy: 80% was deemed to be clearly acceptable, 60–80% a ‘grey area’ and <60% clearly unacceptable. The study was designed to test the null hypothesis that the completion rate was ⩽60% against the alternative of >60% using Fleming's test (Fleming, 1982). Setting a one-sided significance level of 5% and a power of 90% for a true completion rate of 80% required 44 patients to be recruited to each arm.

The study was not powered to detect differences in efficacy between the three arms; randomisation was utilised to ensure that patients with similar characteristics entered each arm. However, it also permitted a preliminary analysis to be made of the range of efficacy to be expected.

Exploratory comparisons of patient characteristics and treatment outcomes between the two arms were carried out using the *χ*^2^ test and Fisher's exact test.

## RESULTS

### Patients

Between June 2001 and September 2002, 100 patients (51 in arm A, 49 in arm B) were recruited from 17 European centres. Demographic characteristics were similar in both groups (Table 2). Most (81%) patients had FIGO stage III–IV disease and 42% had bulky residual disease (>2 cm diameter) after surgery. The most common histological subtype was serous adenocarcinoma and 64% of tumours were moderately or poorly differentiated. Although more patients in arm A were of performance status 0, this difference was not statistically significant (*χ*^2^=3.39, *P*=0.066). Two patients who commenced therapy were subsequently withdrawn owing to incorrect histological diagnosis (one mixed mullerian tumour, one metastatic carcinoid). These patients were included in the toxicity and dose intensity analyses, but excluded from efficacy assessments.

### Chemotherapy administration and dose intensity

**Figure 1:**
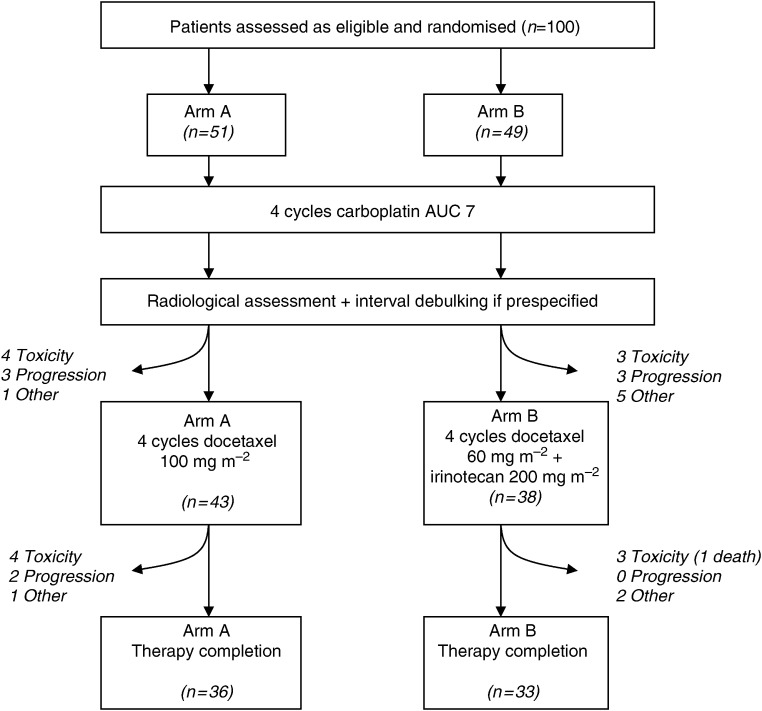
Flow chart indicating progress of the two patient cohorts through Arms A and B. All chemotherapy cycles were administered at 3-weekly intervals. Numbers of patients (*n*) at each stage of therapy are listed. Reasons for discontinuation are given in italics.

A summary diagram indicating the progress of the two patient cohorts through treatment is given in Figure 1.

All patients received ⩾1 cycle of carboplatin AUC 7 and 88 out of 100 patients completed carboplatin therapy. Seventy-seven of 379 carboplatin cycles (20.3%) were delayed by ⩾1 week and 27 cycles were subsequently given at reduced dose. The majority (87%) of the delays were due to haematological toxicity. The median carboplatin dose intensity achieved was 91.7% of the planned dose in arm A and 85.9% in arm B.

Forty-three patients (84%) in arm A commenced docetaxel chemotherapy and 36 (71%; 90% confidence interval (CI) 58–81%) completed all planned therapy (primary end point). Nineteen initial cycles of docetaxel were delayed owing to carboplatin-induced haematological toxicity. In contrast, only six subsequent cycles were delayed by ⩾1 week and, although 14 cycles were dose-reduced (primarily owing to neutropenic fever), the median-dose intensity achieved was 97.7%. Thirty-eight patients (78%) in arm B commenced docetaxel–irinotecan treatment and 33 (67%; 90% CI 55–78%) completed planned therapy (primary end point). Fifteen initial cycles were delayed owing to carboplatin-related toxicity. Twelve subsequent cycles were delayed and 17 given at reduced dose (overall, nine cycles were dose-reduced owing to neutropenic fever and seven owing to gastrointestinal toxicity (primarily diarrhoea)). Of the planned dose, the median-dose intensity was 92.6% for docetaxel and 92.4% for irinotecan.

Neither arm demonstrated a treatment completion rate statistically greater than 60%, which was the predefined feasibility criterion.

### Toxicity

The predominant toxicities reported were haematological (Table 3). As no significant toxicity differences were noted between arms A and B during the carboplatin treatment phase, these data were combined: 10.8% of carboplatin cycles were complicated by grade 3–4 neutropenia and 8.2% by grade 3–4 thrombocytopenia. However, only 2.1% of cycles were complicated by neutropenic fever and 1.1% by thrombocytopenia-related bleeding. Apart from vomiting (grade 3–4 in 5.8% of cycles), non-haematological toxicities were generally mild throughout carboplatin treatment.

During the docetaxel-based treatment phase, differences in toxicity profile were evident between the two arms. Grade 3–4 neutropenia was reported in 63.5 and 50.2% of cycles in arms A and B, respectively; neutropenic fever complicated 7.5 and 5.7% of cycles, respectively. The incidences of other haematological toxicities were low in both arms. Gastrointestinal toxicity was more common in arm B, with grade 3–4 diarrhoea seen in 3.5% of cycles compared with only 0.6% in arm A. Sensory neurotoxicity was reported more frequently in arm A, consistent with the higher docetaxel dose; 5.0% of cycles in arm A *vs* 1.4% in arm B were complicated by grade 2–4 sensory neuropathy (1.9 *vs* 0% by grade 3–4 sensory neuropathy). The differences in toxicity were reflected in the reasons for dose reduction or delay noted above. Grade 2 alopecia was reported in 43.7 and 50.4% of cycles in arms A and B, respectively. Overall, 14 patients (eight in arm A, six in arm B) discontinued protocol treatment because of toxicity; the reasons are listed in Table 4.

Two deaths due to adverse events were reported (both in arm B): one patient died from enterococcal septicaemia and pneumonia associated with grade 4 neutropenia 5 days after receiving docetaxel and irinotecan and one patient died from secondary complications due to a treatment-unrelated ischaemic stroke suffered during the carboplatin treatment phase. At the time of death, this latter patient had discontinued protocol treatment owing to disease progression.

### Efficacy

At baseline, 30 patients in arm A and 25 in arm B had radiologically evaluable disease. Of these, 17 (57%) in arm A achieved a disease response after the carboplatin phase, compared with 12 (48%) in arm B (Table 5). After completion of docetaxel-based treatment, seven patients (23%) in arm A had achieved a complete response and eight (27%) had achieved a partial response; seven (23%) patients had disease progression. In arm B, two patients (8%) achieved a complete response and 10 (40%) achieved a partial response; seven (28%) patients had disease progression.

The change in cytotoxic agents after the first four cycles allowed a preliminary assessment of the relative efficacies of docetaxel *vs* docetaxel–irinotecan in improving disease status. Of the 22 patients in arm A and 19 patients in arm B with partial response or stable disease after carboplatin initiation therapy, six (27%) had improved radiological findings after docetaxel (arm A) compared with three (16%) after docetaxel–irinotecan (arm B) (odds ratio=0.5; 95% CI 0.147–1.702). Disease progression was seen in three (14%) docetaxel patients and three (16%) docetaxel–irinotecan patients.

Although nine patients (9%) had disease progression after carboplatin, only three (3%) remained on protocol. Only one of these patients (arm B) responded to further therapy. Of the patients with radiologically unevaluable disease at trial entry, two patients (one in each arm) experienced disease progression during docetaxel-based therapy.

Sixty-one patients (61%) had disease that could be assessed for CA-125 response using the Gynecologic Cancer Intergroup (GCIG) criteria (Rustin *et al*, 2004). Forty-one of these patients (67%) met the criteria for response, 33 of whom had radiologically measurable disease. Of these patients, 27 had a radiological disease response, five had stable disease and one had radiological disease progression. Of the patients whose CA-125 failed to respond to therapy and who had radiologically measurable disease (11 patients), three had a disease response on cross-sectional imaging.

The median follow-up period was 17.1 months, during which time 49 patients (50%) experienced disease progression. Median progression-free survival was 17.1 months in arm A (95% CI 11.7–22.4 months) and 15.9 months in arm B (95% CI 8.0–23.9 months).

### Quality of life

Of the eligible patients, 82% completed the QoL questionnaires at baseline (77% after four cycles and 71% after eight cycles). However, only 42% of eligible patients completed questionnaires at the first post-treatment follow-up. No significant differences were seen between the two arms in overall change in QoL or change in any symptom-specific subscales.

## DISCUSSION

This randomised phase II study assessed the feasibility of delivering sequential schedules of carboplatin, docetaxel and irinotecan as first-line therapy in advanced EOC. One hundred patients were randomised to receive four 3-weekly cycles of either docetaxel 100 mg m^−2^ (arm A) or docetaxel 60 mg m^−2^ and irinotecan 200 mg m^−2^ (arm B) after four cycles of carboplatin at a dose of AUC 7 every 3 weeks. Both arms just failed to meet the predefined feasibility criterion of a treatment completion rate statistically greater than 60% (arm A 71%, 90% CI 58–81%, *P*=0.079; arm B 67%, 90% CI 55–78%, *P*=0.184).

The overall response rates achieved in patients with radiologically evaluable disease of 50% in arm A and 48% in arm B appear modest for a phase II study in this patient group, despite a median dose intensity of >85% for all drugs administered. The results, however, are consistent with the 58.7% achieved with carboplatin–docetaxel in the SCOTROC I trial on which this study was based (Vasey *et al*, 2004). Importantly, the median progression-free survival figures of 17.1 and 15.9 months in arms A and B, respectively, are also similar to those previously reported in phase II and III studies of first-line chemotherapy in patients with predominantly stage III–IV EOC (International Collaborative Ovarian Neoplasm Group, 2002; Guppy *et al*, 2004; Harries *et al*, 2004; Vasey *et al*, 2004), providing preliminary evidence that the regimens we tested have efficacy consistent with current first-line therapies.

The use of sequential therapy also allowed a preliminary assessment of the relative efficacies of docetaxel and docetaxel–irinotecan in improving disease status after single-agent carboplatin. Patients with persistent disease after carboplatin treatment represented a cohort of patients in this trial with relatively chemoresistant disease. In this cohort, 27% of patients receiving docetaxel alone had improved disease status compared with 16% of patients receiving docetaxel–irinotecan (odds ratio=0.5; 95% CI 0.147–1.702). These figures are consistent with equivalence between the two treatment arms in a study of this size (Fisher's exact test *P*=0.62). They are, however, lower than the response rates reported in a Finnish phase II study of docetaxel–irinotecan in relapsed EOC, which documented a response rate of 63% in both platinum-sensitive and refractory disease (Mäenpää *et al*, 2002). However, Guppy *et al* (2004) reported decreasing response rates for each agent in their small phase II study using sequential carboplatin, paclitaxel and topotecan in a similar patient group to that investigated here.

The present study did reveal differences in the toxicity profiles of the two treatment regimens under investigation, although the toxicities seen were, in general, predictable and manageable. Myelosuppression was the most frequent toxicity (10.8% of carboplatin cycles, 63.5% of docetaxel cycles and 50.2% of docetaxel–irinotecan cycles were associated with grade 3–4 neutropenia), although the rates of complicated neutropenia were low. Differences were also seen with non-haematological toxicity. Significant sensory neuropathy was more common with docetaxel alone (probably secondary to the higher docetaxel dose) whereas serious gastrointestinal side effects were more frequent with docetaxel–irinotecan. A total of 3.5% of cycles of combination chemotherapy were complicated by grade III/IV diarrhoea compared with 0.9% of those using docetaxel alone, and one patient in arm B died of enterococcal septicaemia. While this toxicity pattern has been reported in other phase II studies of docetaxel–irinotecan combinations, it is generally considered acceptable and is not affected by regimen scheduling (Kurtz *et al*, 2003; Pectasides *et al*, 2005; Wachters *et al*, 2005). While such differences were apparent using conventional toxicity scores, no significant QoL differences emerged (either on global measures or on the symptom-specific subscales). Although the EORTC-QLQ30 score has previously been reported to correlate well with toxicity scores for ovarian cancer patients undergoing chemotherapy (Butler *et al*, 2004), the low number of severe objective toxicities and the relatively poor compliance with questionnaire completion may explain the inability of this modality to detect differences in this study.

During the carboplatin initiation phase, seven patients (7%) withdrew owing to excessive toxicity and, subsequently, four patients (9.3%) receiving docetaxel and three (7.9%) receiving docetaxel–irinotecan discontinued therapy owing to toxicity, giving a total withdrawal rate of 14%. These figures compare favourably with those seen in a phase II study of the triplet combination carboplatin AUC 5/paclitaxel 150 mg m^−2^/irinotecan 100 mg m^−2^ administered every 3–4 weeks, where 27% of patients withdrew owing to toxicity (Escobar *et al*, 2004).

Most previous studies investigating the incorporation of topoisomerase-I inhibitors into first-line therapy of EOC have used triplet combinations, with subsequent difficulties in dose escalation owing to myelotoxicity (Herben *et al*, 1999; Cacciari *et al*, 2000; Bolis *et al*, 2001; Escobar *et al*, 2004). The results reported here indicate the feasibility of this concept by using a sequential approach to maintain dose intensity. In particular, a similar total dose of carboplatin (AUC 28 *vs* 30) was administered compared to standard carboplatin–paclitaxel regimens and a higher dose intensity (AUC 2.5 *vs* 1.25 per week) was achieved during the platinum phase of therapy. We also documented a manageable toxicity profile for docetaxel–irinotecan that reduces neurotoxicity compared to using a higher dose of docetaxel alone although at the expense of increased diarrhoea. However, the recent results of two randomised phase III studies (De Placido *et al*, 2004; Pfisterer *et al*, 2005) have failed to demonstrate an improvement in survival with the addition of four cycles of the topoisomerase-I inhibitor topotecan after standard carboplatin–paclitaxel as first-line treatment for EOC. Although the MITO-1 study (De Placido *et al*, 2004) can be criticised for only effectively assessing the concept of consolidation therapy, as 87% of patients randomised to topotecan or placebo had already obtained a complete response to standard treatment, patients were randomised at registration in the GINECO/AGO-OVAR trial (Pfisterer *et al*, 2005) and 78% of those randomised to sequential topotecan received this therapy.

Nevertheless, the present study suggests that further investigation of the concept of delivering sequential combinations of carboplatin, docetaxel and irinotecan at high-dose intensity to patients with ovarian cancer may be appropriate.

## Figures and Tables

**Table 1:** Dose levels after reduction for toxicity

**Study arm**	**Dose level 0**	**Dose level 1**	**Dose level 2**
Carboplatin	AUC 7	AUC 6	AUC 5
Docetaxel (arm A)	100 mg m^−2^	75 mg m^−2^	60 mg m^−2^
Docetaxel–irinotecan (arm B)	Docetaxel: 60 mg m^−2^; irinotecan: 200 mg m^−2^	25% reduction with both drugs	Further 25% reduction with both drugs

AUC=area under the curve.

**Table 2:** Patient demographics

**Age**	**Arm A (*n*=51)**	**Arm B (*n*=49)**
Median	56	60
Range	31–72	41–76

**Performance status**	**No. of patients**	**%**	**No. of patients**	**%**
0	39	76.5	28	57.1
1	11	21.6	21	42.9
2	1	1.9	0	0
*FIGO stage*				
Ic	4	7.8	0	0
II	7	13.7	8	16.3
III	37	72.6	36	73.5
IV	3	5.9	5	10.2
*Residual disease*				
None/micro	13	25.5	13	26.5
⩽2 cm	17	33.3	15	30.6
>2 cm	21	41.2	21	42.9
*Histological subtype*				
Serous/papillary	24	47.1	28	57.1
Mucinous	4	7.8	2	4.1
Clear cell	3	5.9	3	6.1
Endometrioid	5	9.8	5	10.2
Other	12	23.5	9	18.3
Not known	3	5.9	2	4.1

FIGO=International Federation of Gynecologic Oncology.

**Table 3:** Percentage of cycles of chemotherapy complicated by toxicity

			**Docetaxel ± irinotecan**			
	**Carboplatin**		**Arm A**		**Arm B**	
**No. of cycles**	**379**		**161**		**141**	
**Grade**	**3**	**4**	**3**	**4**	**3**	**4**
*Haematological toxicity*						
Neutropenia	8.6	2.2	22.3	41.2	25.2	25.0
Anaemia	4.1	0.5	0	0.7	0	0
Thrombocytopenia	5.5	2.7	0	0	0.8	0.8
*Gastrointestinal*						
Nausea	1.1	—	0.6	—	2.8	—
Vomiting	4.7	1.1	1.9	0	1.4	0
Diarrhoea	1.1	0.3	0.6	0	2.8	0.7
Constipation	0.8	0	0	0	0.7	0
Abdominal pain	1.3	0	0	0	0.7	0.7
*Neurological*						
Sensory	0.3	0	1.9	0	0	0
Motor	0	0	0	0	0	0
*Other*						
Oedema	0.3	0	0	0	0	0
Dyspnoea	0.3	0.3	0	0	0	0
Stomatitis	0.5	0	0.6	0	0	0

**Table 4:** Reasons for treatment discontinuation

	**Arm A**					**Arm B**				
**Cycle**	**No. of patients completed**	**Toxicity**	**Progression**	**Consent**	**Other**	**No. of patients completed**	**Toxicity**	**Progression**	**Consent**	**Other**
1	51	—	—	—	—	49	—	—	—	—
2	49	1	1	0	0	48	1	0	0	0
3	48	2	1	0	0	46	2	1	0	0
4	45	4	2	0	0	43	3	2	1	0
5	43	4	3	1	0	38	3	3	2	3
6	42	5	3	1	0	35	4	3	4	3
7	40	5	5	1	0	35	4	3	4	3
8	36	8	5	1	1	33	6	3	4	3

**Table 5:** Radiological response assessment in patients with evaluable disease at trial entry

	**Arm A (*n*=30)**		**Arm B (*n*=25)**	
	**Post-carboplatin**	**Overall**	**Post-carboplatin**	**Overall**
CR	3 (10)	7 (23)	0 (0)	2 (8)
PR	14 (47)	8 (27)	12 (48)	10 (40)
CR + PR	17 (57)	15 (50)	12 (48)	12 (48)
SD	8 (27)	5 (17)	7 (28)	4 (16)
PD	4 (13)	7 (23)	5 (20)	7 (28)
Measurable disease^a^	1 (3)	3 (10)	1 (4)	2 (8)
Non-evaluable	20 (—)		23 (—)	

a Failed to complete therapy.

Values in parentheses are percentages given as the proportion of patients with radiologically evaluable disease at trial entry.

CR=complete response; PD=progressive disease; PR=partial response; SD=stable disease.
